# Placental characteristics and neonatal weights among women with malaria-preeclampsia comorbidity and healthy pregnancies

**DOI:** 10.1371/journal.pone.0291172

**Published:** 2023-10-19

**Authors:** Everett Lwamulungi, Zahida Qureshi, Moses Obimbo, Omondi Ogutu, Eunice Cheserem, Rose J. Kosgei, Edwin Walong, Dennis Inyangala, George G. Nyakundi, Patrick M. Ndavi, Alfred O. Osoti, Diana K. Ondieki, Anne N. Pulei, Anne Njoroge, Sarah Masyuko, Cyrus M. Wachira

**Affiliations:** 1 Department of Obstetrics and Gynecology, University of Nairobi, Nairobi, Kenya; 2 Kenyatta National Hospital, Nairobi, Kenya; 3 Department of Human Anatomy, University of Nairobi, Nairobi, Kenya; 4 Department of Pathology, University of Nairobi, Nairobi, Kenya; 5 Department of Global Health, University of Washington, Seattle, Washington, United states of America; Bangabandhu Sheikh Mujib Medical University (BSMMU), BANGLADESH

## Abstract

**Background:**

Malaria and preeclampsia are leading causes of maternal morbidity and mortality in sub-Saharan Africa. They contribute significantly to poor perinatal outcomes like low neonatal weight by causing considerable placental morphological changes that impair placental function. Previous studies have described the effects of either condition on the placental structure but the structure of the placenta in malaria-preeclampsia comorbidity is largely understudied despite its high burden. This study aimed to compare the placental characteristics and neonatal weights among women with malaria-preeclampsia comorbidity versus those with healthy pregnancies.

**Methodology:**

We conducted a retrospective cohort study among 24 women with malaria-preeclampsia comorbidity and 24 women with healthy pregnancies at a County Hospital in Western Kenya. Neonatal weights, gross and histo-morphometric placental characteristics were compared among the two groups.

**Results:**

There was a significant reduction in neonatal weights (P<0.001), placental weights (P = 0.028), cord length (P<0.001), and cord diameter (P<0.001) among women with malaria-preeclampsia comorbidity compared to those with healthy pregnancies. There was also a significant reduction in villous maturity (P = 0.016) and villous volume density (P = 0.012) with increased villous vascularity (P<0.007) among women with malaria-preeclampsia comorbidity compared to those with healthy pregnancies.

**Conclusion:**

Placental villous maturity and villous volume density are significantly reduced in patients with malaria-preeclampsia comorbidity with a compensatory increase in villous vascularity. This leads to impaired placental function that contributes to lower neonatal weights.

## Introduction

Malaria and preeclampsia frequently occur together in the tropics and cause significant fetal and maternal adverse effects [1]. Globally, malaria and hypertensive disorders in pregnancy, including preeclampsia account for 86,000 maternal and 700,000 perinatal deaths annually [2, 3]. The two conditions individually cause significant alterations of placental structure [4–9]. Placental malaria causes a reduction in placental weight, excess fibrin deposition, trophoblastic necrosis, basement membrane thickening, reduced villous size, villous perimeter and villous vascularity [4–6]. Preeclampsia causes reduced placental weight, increased syncytial knotting, fibrin deposition, necrosis of placental discs, spiral artery hypertrophy, villous hypoplasia and villous thrombosis [7–9].

Furthermore, preeclampsia and malaria cause placental inflammation that disrupts physiological functionality [10]. In preeclampsia, placental hypoxic stress results from reduced placental perfusion emanating from inadequate spiral artery invasion by extra-villous trophoblasts. Consequently, this triggers release of proinflammatory cytokines and antiangiogenic factors which further undermine placental development [11]. Placental malaria similarly causes increased cytokine production and inflammatory cell infiltration resulting in oxidative stress induced damage to placental cells [12]. These blemishes caused by either condition individually and jointly contribute to prematurity, low birth weight and fetal demise [13, 14].

Earlier studies have shown a high burden of malaria-preeclampsia comorbidity occurring in malaria endemic areas of the tropics [15]. The placental structure has many implications on maternal, perinatal and neonatal outcomes such as low birth weight and fetal demise [16]. However, the structure of the placenta in malaria-preeclampsia comorbidity is underexplored. Understanding the placental structure in this comorbidity is critical in understanding the pathophysiology of the associated maternal, fetal and neonatal adverse outcomes. This may inform specific therapeutic remedies to mitigate these complications in settings like Western Kenya where both conditions are highly prevalent.

We conducted a retrospective cohort study to compare the placental characteristics and neonatal weights among women with malaria-preeclampsia comorbidity and those with healthy pregnancies at Bungoma County Referral Hospital.

## Methodology

### Study design

This was a retrospective cohort study in which the exposed group was women with malaria-preeclampsia comorbidity and the unexposed group was women with healthy pregnancies. The outcome was placental characteristics and neonatal weights.

### Study site and setting

The study site was Bungoma County Referral Hospital which is located in the lake endemic malaria zone of Kenya about 400kms west of the capital, Nairobi. The county has a malaria prevalence of 27% in the general population and 21.6% among pregnant women with a maternal mortality rate of 382 per 100,000 live births which is one of the highest in the country. A diagnosis of malaria was based on evidence of parasitemia on blood film. Preeclampsia was diagnosed based on evidence of new onset hypertension greater or equal to 140/90mmHg at or after the 20th gestation week with proteinuria on dipstick or evidence of end organ damage. Malaria in pregnancy at this hospital is managed with oral or intravenous antimalarial medication. Preeclampsia is managed with antihypertensive medication to control hypertension, magnesium sulphate for seizure prophylaxis and end organ damage management. The women included in this study had their placentae collected and stored in the biorepository at the Basic, Clinical and Translational (BCT) research laboratory for purposes of future research. The placental collection was done immediately following delivery from January 2018 to December 2019. The maternal clinical characteristics, neonatal weights and gross placental characteristics were also recorded at the time of placental collection. The BCT laboratory is located at the Department of Human Anatomy at the University of Nairobi.

### Study participants

We included singleton pregnancies, women with complete medical records, term gestation and those aged between 18 to 49 years. We excluded women with preexisting cardiovascular disease; chronic hypertension; diabetes; infections like HIV and hepatitis; and women with a history of cigarette smoking.

### Sample size and sampling procedure

**Fig 1:**
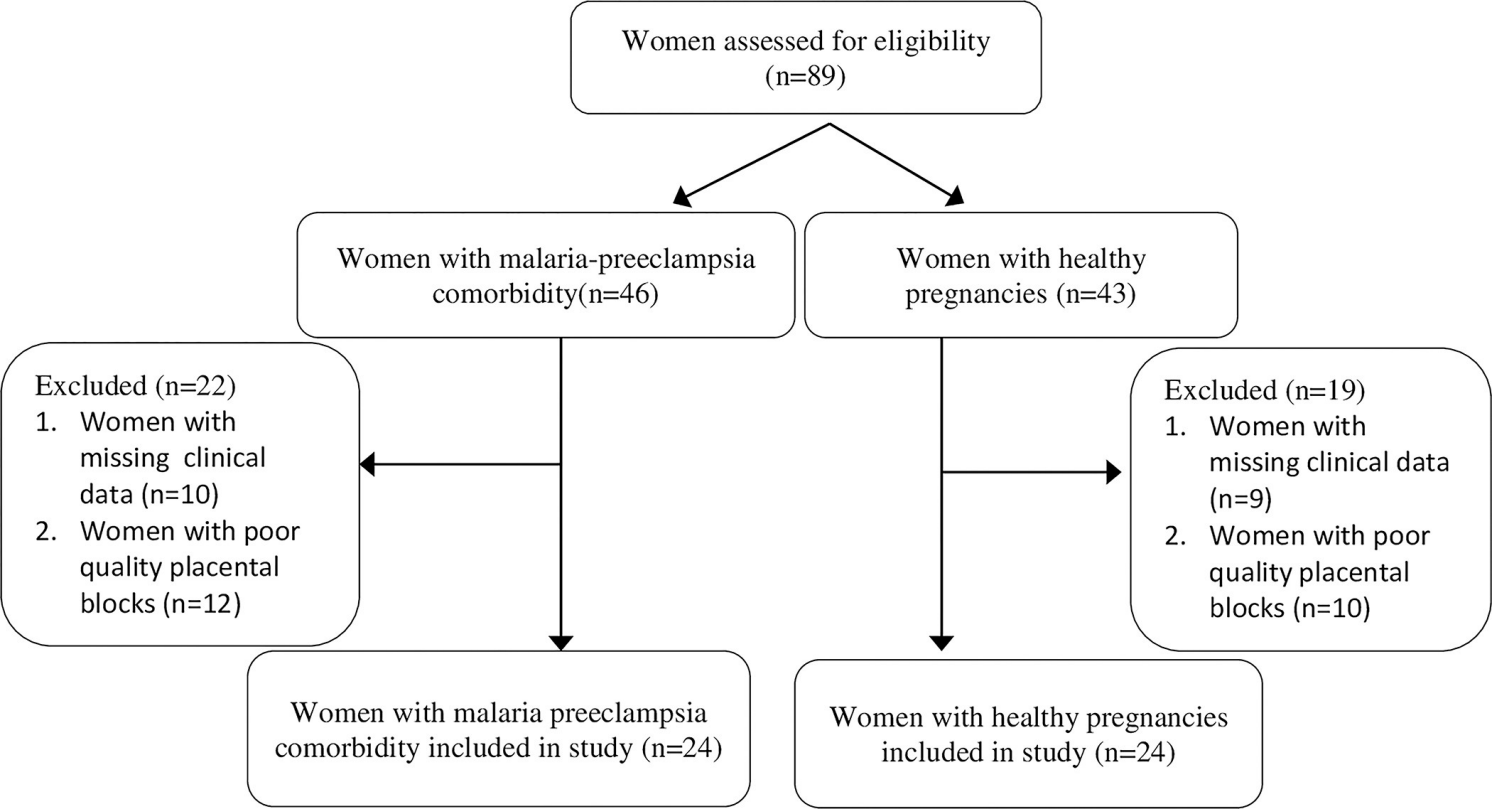
Participant selection.

The total calculated sample size using Kelsey’s formula was 48 with 24 participants per group [17]. A total of 89 women with malaria-preeclampsia comorbidity and healthy pregnancies that had term deliveries were assessed for eligibility for inclusion in the study. Forty one (46%) were excluded (15 due to missing clinical data and 26 due to poor quality placental blocks). The excluded paraffin blocks had inadequate fixation or inappropriate processing. Forty eight (54%) were included in the study (Fig 1).

### Study procedure

#### Gross examination

The following placental characteristics were measured at the primary placental collection and recorded in the placental pathology form: weight of the placenta, gestational age, presence of areas of infarction or thrombosis, shape of the placental specimen, the colour of the placental membranes and the chorionic plate, length of the umbilical cord and mean diameter of the cord.

#### Microscopic evaluation of villous tree and basal plate

**Fig 2:**
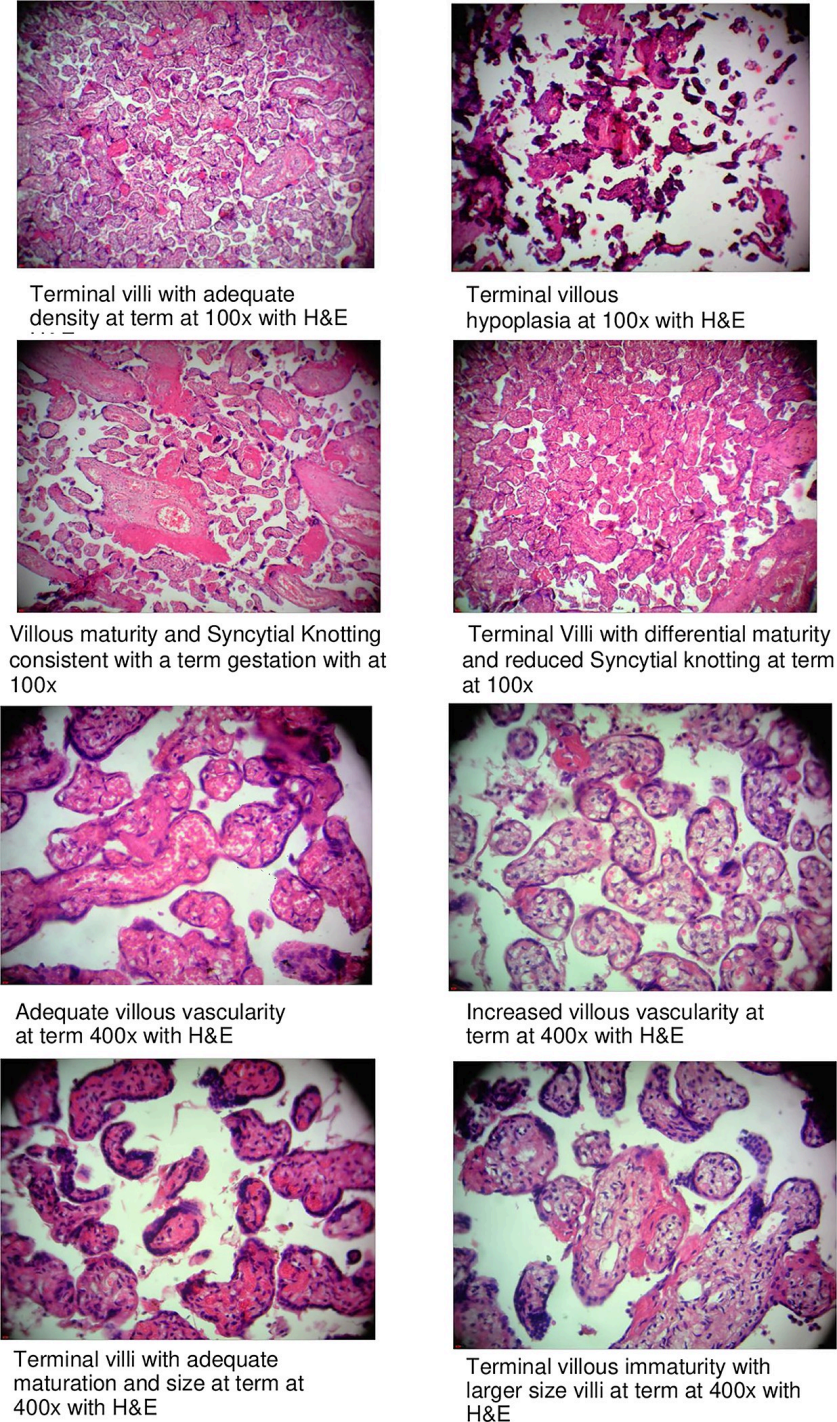
Select micrographs of placentae with features of reduced villous maturity and increased vascularity.

Consecutive sections of placental specimens measuring seven micrometers in thickness were prepared using a Leitz Wetzlar sledge microtome. These were floated in warm water and fixed on glass slides. Drying in an oven at 40°C was then done overnight. The serial sections were stained with Masson’s trichrome, or hematoxylin and eosin (H&E). H&E was used for demonstrating the general histomorphometry and Masson’s trichrome was used to exhibit the connective tissue constituents. The slides were then analyzed using a Richter Optica Plan Achromatic UX-1T digital light microscope interphased with a Moticam BTU10 Camera system then connected to a computer and monitor (Fig 2). The team of examiners comprised a placental biologist, two anatomists, and two pathologists who examined the placentae individually after which they summarized the findings collectively. The examiners were blinded to the status of the disease of the mothers. The overall histology of the amnion and chorion was recorded together with the degree of deposition of fibrin. Other characteristics that were graded as described by the Fisher group are degeneration of villi, delamination of the syncytiotrophoblast, villous vascularity, adhesion of red blood cells to the terminal villi, maturity of villi, syncytial knotting, villitis, and deciduitis [18].

#### Morphometric analyses of terminal chorionic villi

The structure of placental terminal villi from either group was further analyzed morphometrically at 400× magnification. The team examined the tissue sections using a Richter Optica Plan Achromatic UX-1T digital light microscope interphased with a Moticam BTU10 Camera system and then connected to a computer and monitor. Digital images were captured using Motic software from ten randomly selected areas from each slide. A calibrated scale was applied to aid in measurement. Data collection was restricted to the terminal villi whose outlines were entirely inside the microscopic field. The images were analyzed using Image J version 2.1. The Feret diameter, perimeter, cross-sectional area and villous density were calculated for each of the terminal villi within the microscopic field and mean values were computed per case. Findings were analyzed following the Astshuler classification [19].

### Data variables

The exposure variables were malaria-preeclampsia comorbidity and healthy pregnancies. The outcome variables were neonatal weights and placental characteristics.

### Data analysis

Placental gross anatomical features, histological features, morphometry of placental terminal villi, maternal clinical characteristics and neonatal weights of women with malaria-preeclampsia comorbidity were compared to those with healthy pregnancies. Quantitative data analysis was conducted using R software. Descriptive analysis was presented using frequency tables and graphs. Mean values and standard deviation were used for continuous variables while frequencies and percentages were used for categorical variables. Inferential statistics for comparison between the two groups was done using a two-sample t-test, Wilcoxon rank sum test, chi-square test and Fisher’s exact test. A p-value of <0.05 was considered statistically significant.

### Ethical considerations

Informed written consent to collect placental specimen and use medical records was obtained from the participants before inclusion in this study. Ethical approval for the collection of the placental specimen and storage at the BCT laboratory was granted by the Mount Kenya University, Ethical and Review Committee (MKU/ERC/0543). For our study, permission to analyze anonymized samples was sought from the Kenyatta National Hospital and the University of Nairobi, Ethical Review Committee (P645/11/2020).

## Results

### Participant characteristics

The mean maternal age among women with malaria-preeclampsia comorbidity was 29 years and 26 years among those with healthy pregnancies. The majority of the women with healthy pregnancies were primi-gravidas while those with the comorbidity were mostly multigravidas. The level of education and marital status were comparable between the two groups (Table 1).

**Table 1:** Demographics, clinical characteristics and neonatal weights among women with malaria-preeclampsia comorbidity and healthy pregnancies at Bungoma County referral hospital in 2018 to 2019.

VARIABLE	Women with malaria-preeclampsia comorbidity (N = 24*)	Women with healthy pregnancies (N = 24*)	P-value**
Age	29 (5.2)	26 (3.7)	0.012
Marital Status			
Married	22 (91.6)	18 (75.0)	0.2
Single	2 (8.3)	6 (25.0)	
Education			>0.9
Primary School	6 (25)	6 (25)	
High School	12 (50)	13 (54)	
College	6 (25)	5 (20)	
Parity			0.004
0–1	7 (29)	17 (70.8)	
2+	17 (70.8)	7 (29.1)	

*Mean (SD); n (%)

**Two sample t-test; Fisher’s exact test; Pearson’s Chi-squared test

### Gross placental characteristics

The mean placental weight of 502g (P-0.028) and mean umbilical cord length of 47cm (P<0.001) were statistically significantly lower in the women with malaria-preeclampsia comorbidity compared to women with healthy pregnancies. The mean cord diameter of 4cm (P<0.001) was statistically significantly higher in the women with comorbidity as compared to those with healthy pregnancies. The placental shape, colour of membranes and presence of areas of infarction were comparable between the two groups (Table 2).

**Table 2:** Gross placental characteristics among women with malaria-preeclampsia comorbidity and healthy pregnancies at Bungoma County referral hospital in 2018 to 2019.

VARIABLE	Women with malaria-preeclampsia comorbidity (N = 24*)	Women with healthy pregnancies(N = 24*)	P-value**
Placental Shape			0.4
Circular	15 (62.5)	12 (50.0)	
Ovoid	9 (37.5)	12 (50.0)	
Placental Weight (g)	502 (55.5)	532 (31.7)	**0.028**
Cord Length (cm)	47 (5.0)	53 (4.8)	**<0.001**
Cord Diameter (cm)	4 (1.1, 1.6)	2 (1.8, 2.0)	**<0.001**
Color of Membranes			0.05
Grey	5 (20.8)	0 (0)	
Maroon	19 (79.1)	24 (100)	
Infarction/thrombosis	7 (38.8)	7 (31.8)	>0.9

* n (%); Mean (SD); Median (IQR)

** Pearson’s Chi-squared test; Wilcoxon rank sum test; Fisher’s exact test; Two- sample t-test

The bolded findings are statistically significant

### Histo-morphometric characteristics

We found a statistically significantly higher incidence of decreased villous maturity of 38% (P-0.016), mean villous cross-sectional area of 1,340 um^2^ (P-0.001), increased villous vascularity of 17% (P-0.007), mean villous perimeter 142 um (P-0.003) and mean villous diameter 48 um (P-0.004) among women with malaria-preeclampsia comorbidity compared to those with healthy pregnancies. There was a statistically significantly lower percentage of syncytial knotting of 46% (P<0.001) and terminal villous volume density of 66% (P-0.012) in the former compared to the latter group. The other parameters we assessed were comparable between the two groups (Table 3).

**Table 3:** Histo-morphometric characteristics among women with malaria-preeclampsia comorbidity and healthy pregnancies at Bungoma County referral hospital in 2018 to 2019.

VARIABLE	Women with malaria-preeclampsia comorbidity (N = 24*)		Women with healthy pregnancies (N = 24*)	P-value**
Distal villous hypoplasia	8 (33.3)		3 (12.5)	0.086
Villous edema	0 (0)		2 (8)	0.5
Villous necrosis	0 (0)		2 (8)	0.5
Syncytial knotting	11 (46)		24 (100)	**<0.001**
Decreased Villous maturity	9 (38)		2 (8)	**0.016**
Thickening of villous BM	0 (0)		4 (17)	0.11
Villitis	2 (8)		0 (0)	0.5
Inter villositis	4 (17)		0 (0)	0.11
Fetal inflammatory response	10 (42)		2 (8)	**0.008**
Villous vascularity	Appropriate	19 (79)	14 (58)	**0.007**
	Decreased	1 (4)	9 (38)	
	Increased	4 (17)	1 (4)	
Mean villous cross sectional area (Um^2^)	1,340 (1,079, 1,607)		678(591, 900)	**0.001**
Mean villous perimeter (Um)	142 (124, 159)		98 (93, 123)	**0.003**
Mean villous diameter (Um)	48 (43, 55)		34 (33, 42)	**0.004**
Mean volume density (%)	66 (48, 73)		76 (72, 79)	**0.012**

* n (%), Median (IQR)

** Fisher’s exact test; Pearson’s Chi-squared test; Wilcoxon rank sum test

The bolded findings are statistically significant

### Neonatal weights

Women with healthy pregnancies had neonates with a significantly higher mean weight of 3,133g compared to those with malaria-preeclampsia comorbidity (2584g, P<0.001).

## Discussion

In this retrospective cohort we found that placentae of women with malaria-preeclampsia comorbidity were associated with features characteristic of villous immaturity like reduced placental weight, larger terminal villous sizes, reduced villous density and reduced syncytial knotting in comparison to placentae of those with healthy pregnancies. We also found a greater percentage of fetal inflammatory response with a compensatory increase in villous vascularity and lower neonatal weights in the comorbidity group in comparison to the healthy pregnancy group.

Our results are similar to previous studies done in women with either malaria or preeclampsia alone that showed low birth weights in pregnancies affected by these conditions [13, 14]. This can be attributed to impaired maternal-fetal circulation due to villous immaturity which causes reduced surface area for nutrient exchange. Immature villi also have a greater maternal-fetal diffusion distance that further compounds the problem [20].

The findings of villous immaturity in our study are, however, in contrast with findings of accelerated villus maturity found by previous scholars in women with malaria or preeclampsia occurring alone [4, 21]. This could imply that when the two conditions occur jointly, they delay placental maturity while they cause accelerated placental maturity individually. The decreased level of villous maturity is possibly due to interference with intrauterine cytokine concentration. Cytokines like placental growth factor (PlGF), vascular endothelial growth factor (VEGF) and soluble Fms-like tyrosine kinase 1 (sFlt-1) are essential for placental development and maturation [22, 23]. Further, immune-histochemical evaluation of the effect of malaria-preeclampsia comorbidity on placental cytokine concentration is needed to confirm this.

To our knowledge, our study was the first in the African region to analyse placental structure in malaria-preeclampsia comorbidity and its association with neonatal weights. This adds to the body of knowledge in this understudied area. It also employed gross, histological and morphometric techniques of placental examination which enabled the comparison of numerous facets of placental structure. Our team of examiners was also blinded to the disease status of the women and the neonatal weights at the time of characterization of placental structure. This prevented observer bias. We were however limited by the relatively small sample size in comparison to previous studies. Our study was also conducted in one hospital and the results may not be generalizable to all settings. Our study did not compare other markers of fetal and neonatal wellbeing like biophysical profile and APGAR scores as they were not universally documented in patient charts.

## Conclusion

In conclusion, placental villous maturity is statistically significantly lower in women with malaria-preeclampsia comorbidity in comparison to those with healthy pregnancies with a compensatory increase in terminal villous vascularity. This leads to impaired placental function that contributes to lower neonatal weights.

## Supporting information

S1 Dataset (ZIP)
