# Supplementary material for: Placental characteristics and neonatal weights among women with malaria-preeclampsia comorbidity and healthy pregnancies
Source: PLoS One. 2023 Oct 19;18(10):e0291172. doi: 10.1371/journal.pone.0291172 (PMC10586625; doi:10.1371/journal.pone.0291172)
Supplement: S1 Dataset — (ZIP) [file pone.0291172.s001.zip › Clinical data.pdf]

Serial numbuer	Study sute	Consent	Age	Parity	Gestational Age
C1	Bungoma	Yes	19	0+0	38
C6	Bungoma	Yes	18	0+0	39
C8	Bungoma	Yes	23	1+1	40
C9	Bungoma	Yes	25	1+2	35
C10	Bungoma	Yes	24	2+0	34
C18	Bungoma	Yes	23	1+0	37
C19	Bungoma	Yes	26	3+1	39
C24	Bungoma	Yes	31	3+0	39
C25	Bungoma	Yes	33	3+1	40
C41	Bungoma	Yes	26	0+1	36
C44	Bungoma	Yes	27	2+0	41
C50	Bungoma	Yes	28	3+1	37
C58	Bungoma	Yes	36	4+0	38
C62	Bungoma	Yes	33	2+1	33
C64	Bungoma	Yes	31	2+2	36
C67	Bungoma	Yes	22	1+1	39
C68	Bungoma	Yes	26	1+1	39
C71	Bungoma	Yes	19	0+1	40
C77	Bungoma	Yes	19	0+0	37
C89	Bungoma	Yes	23	2+0	37
CC1	BUNGOMA	YES	21	1+0	41
CC2	BUNGOMA	YES	23	2+1	39
CC3	BUNGOMA	YES	21	1+1	40
CC4	BUNGOMA	YES	25	1+0	38
CC5	BUNGOMA	YES	24	0+0	38
CC6	BUNGOMA	YES	23	1+1	41
CC7	BUNGOMA	YES	25	0+2	40
CC8	BUNGOMA	YES	26	1+0	40
CC9	BUNGOMA	YES	27	2+0	40
CC10	BUNGOMA	YES	22	0+1	38
CC11	BUNGOMA	YES	24	1+0	40
CC12	BUNGOMA	YES	32	3+1	39
CC13	BUNGOMA	YES	30	2+1	40
CC14	BUNGOMA	YES	34	3+0	38
CC15	BUNGOMA	YES	33	1+1	40
CC16	BUNGOMA	YES	26	0+1	39
CC17	BUNGOMA	YES	27	1+1	38
CC18	BUNGOMA	YES	25	1+1	40
CC19	BUNGOMA	YES	35	2+2	41
CC20	BUNGOMA	YES	23	1+1	40
CC21	BUNGOMA	YES	22	1+2	37
CC22	BUNGOMA	YES	26	1+2	38
CC23	BUNGOMA	YES	28	2+1	40
CC24	BUNGOMA	YES	27	1+0	37
CC25	BUNGOMA	YES	30	3+0	40
c163	BUNGOMA	YES	26	0+1	33
c154	BUNGOMA	YES	23	1+1	34

c155	BUNGOMA	YES	31	1+1	33
c183	BUNGOMA	YES	33	3+0	32
c171	BUNGOMA	YES	26	2+0	36
c173	BUNGOMA	YES	25	1+2	36
cc41	BUNGOMA	YES	24	1+0	36
c156	BUNGOMA	YES	29	1+0	37
cc44	BUNGOMA	YES	31	1+1	36
c152	BUNGOMA	YES	31	2+3	36
c161	BUNGOMA	YES	21	0+0	35
cc49	BUNGOMA	YES	36	4+0	33
c163	BUNGOMA	YES	34	2+2	34
cc42	BUNGOMA	YES	35	3+1	34
cc44	BUNGOMA	YES	27	2+1	36
cc50	BUNGOMA	YES	26	1+1	37
c185	BUNGOMA	YES	28	2+1	37
cc29	BUNGOMA	YES	29	1+1	37
cc31	BUNGOMA	YES	27	3+0	33
cc46	BUNGOMA	YES	33	3+0	36
cc32	BUNGOMA	YES	34	1+0	35
cc34	BUNGOMA	YES	27	2+1	35
cc36	BUNGOMA	YES	27	1+1	37
cc37	BUNGOMA	YES	28	2+2	36
c175	BUNGOMA	YES	33	3+1	33
cc39	BUNGOMA	YES	31	3+0	35
cc37	BUNGOMA	YES	23	1+0	36
cc51	BUNGOMA	YES	24	1+0	35
cc56	BUNGOMA	YES	28	2+1	37
c173	BUNGOMA	YES	34	3+1	37
cc30	BUNGOMA	YES	33	2+1	36
cc28	BUNGOMA	YES	37	4+0	34
cc35	BUNGOMA	YES	34	3+1	35
cc43	BUNGOMA	YES	27	1+1	36
cc47	BUNGOMA	YES	33	2+0	37

Marital statu	Education	Hb	Placental weight	Neonatal weight	Placental shape
Married	High school	12.1	440	2810	ovoid
Single	Primary scho	11.9	470	2750	circular
Married	High school	10.8	530	3630	circular
Married	High school	11.3	420	2940	circular
Married	Primary scho	12	430	2840	ovoid
Single	Primary scho	9	490	2650	ovoid
Single	High school	10.1	550	3310	ovoid
Married	Primary scho	10.6	600	3830	circular
Married	Primary scho	9	580	3450	circular
Married	College	11.1	490	3110	circular
Married	High school	10.3	580	3250	circular
Married	High school	11.6	510	3110	circular
Married	Primary scho	10.9	530	3420	circular
Married	College	9.8	420	2590	circular
Married	High school	10.2	480	2980	circular
Single	High school	10.1	580	3330	circular
Married	Primary scho	9.9	610	3610	circular
Single	High school	10.4	550	3120	circular
Married	High school	11.4	530	3410	circular
Married	Primary scho	10.8	500	2970	circular
MARRIED	Primary scho	11.3	520	3400	Ovoid
MARRIED	High school	10.7	540	3320	Circular
SINGLE	High school	13.8	600	3210	Ovoid
MARRIED	Primary scho	13.1	510	3300	Circular
MARRIED	High school	10	500	2850	Ovoid
SINGLE	Primary scho	11.9	530	3330	Circular
MARRIED	college	10.7	580	3830	Ovoid
MARRIED	Primary scho	11.8	490	3110	Ovoid
MARRIED	College	11	500	2910	Circular
SINGLE	Primary scho	14.1	500	2700	Ovoid
MARRIED	High school	13.1	570	3020	Circular
SINGLE	High school	14.7	580	3400	Ovoid
MARRIED	Primary scho	10.7	480	2440	Circular
MARRIED	High school	11.4	600	3760	circular
MARRIED	High school	12.1	550	3090	Circular
MARRIED	High school	13.1	540	2900	Ovoid
SINGLE	High school	10.3	540	3000	Ovoid
MARRIED	College	15.4	560	3120	Ovoid
MARRIED	High school	12.3	550	3330	Circular
MARRIED	college	11.4	490	2800	Circular
MARRIED	High school	12.1	490	2930	Ovoid
MARRIED	High school	10	510	3020	Circular
MARRIED	college	9.9	520	3310	Circular
MARRIED	High school	11	530	3060	Circular
MARRIED	High school	13.2	500	2910	Ovoid
MARRIED	HIGH SCHOO	10.2	430	2310	Circular
SINGLE	College	NIL	410	2220	Circular

MARRIED	Primary	10.6	440	1980	ovoid
MARRIED	Primary	10.3	390	1940	Circular
MARRIED	High school	11	510	2360	Circular
SINGLE	primary	12.1	550	2230	Ovoid
MARRIED	high school	10.4	530	2400	ovoid
MARRIED	high school	12	550	2440	Circular
MARRIED	high school	11.1	510	2610	Circular
MARRIED	high school	11.3	520	2550	Circular
SINGLE	primary	11.4	490	2100	ovoid
MARRIED	primary	10.9	470	1980	Circular
MARRIED	college	10.5	510	2090	ovoid
MARRIED	college	10.3	510	2180	Circular
MARRIED	primary	9.8	530	2050	ovoid
SINGLE	high school	10.2	580	2890	ovoid
MARRIED	high school	11.2	570	2760	Circular
MARRIED	high school	11.2	540	2430	Circular
SINGLE	high school	14	450	1890	Circular
MARRIED	primary	12.1	490	2170	Circular
MARRIED	primary	11.4	480	2090	Circular
SINGLE	college	11.5	470	1950	Circular
MARRIED	high school	10.3	560	3010	ovoid
MARRIED	high school	10.4	470	2830	ovoid
MARRIED	high school	9.7	410	1880	ovoid
MARRIED	high school	9.9	460	2350	Circular
MARRIED	high school	10.2	480	2190	Circular
MARRIED	high school	10.4	490	2090	Circular
MARRIED	high school	11.2	510	2340	ovoid
MARRIED	college	11.7	560	2470	Circular
MARRIED	college	12	580	2860	ovoid
MARRIED	college	10	450	2100	ovoid
MARRIED	college	11.3	480	2060	Circular
SINGLE	high school	11.9	550	2150	Circular
MARRIED	high school	10.5	540	2340	ovoid

Cord length (	Cord diama	Colour of me	Infarction/Thrombosis
38	1.1	Maroon	Yes
44	1.2	Maroon	Yes
46	1.4	Maroon	No
52	1.1	Maroon	No
55	1	Maroon	No
39	1.6	Maroon	Yes
46	1.5	Maroon	Yes
44	1.4	Maroon	Yes
47	1.3	Maroon	Yes
54	1.3	grey	No
51	1.9	Maroon	Yes
52	1.7	Maroon	No
43	1.8	Maroon	Yes
44	1	grey	No
47	1.2	Maroon	Yes
36	2	Maroon	Yes
45	2	Maroon	No
47	2	Maroon	Yes
51	1.8	Maroon	Yes
44	1.7	Maroon	Yes
52	1.9	Maroon	No
62	1.8	Maroon	Yes
55	1.7	Maroon	No
54	1.9	Maroon	No
55	2	Maroon	Yes
55	2	Maroon	No
49	2.2	Maroon	No
51	2.1	Maroon	No
50	1.8	Maroon	No
56	2	Maroon	Yes
44	2	Maroon	No
49	2	Maroon	No
50	1.9	Maroon	No
52	1.9	Maroon	No
47	1.7	Maroon	Yes
48	1.6	Maroon	No
61	1.8	Maroon	No
60	1.9	Maroon	No
55	1.8	Maroon	Yes
56	1.6	Maroon	No
58	1.5	Maroon	No
46	2.3	Maroon	Yes
56	2.2	Maroon	No
48	2.1	Maroon	Yes
53	1.9	Maroon	No
51	1.2	Maroon	No
43	1.1	Grey	Yes

44	1.3	Maroon	No
46	1.1	Grey	Yes
53	1	Maroon	No
44	1.2	Maroon	No
45	1.2	Maroon	No
52	1.2	Maroon	No
54	1.1	Maroon	No
53	1.4	grey	Yes
44	1.3	Maroon	Yes
46	1.5	Maroon	No
46	1.4	Maroon	Yes
45	1.1	Maroon	No
39	1.1	Grey	No
45	1	Maroon	No
44	1	Maroon	YES
36	1	Maroon	No
51	1.3	Maroon	No
52	1.6	Maroon	No
47	1.7	Maroon	No
45	1.1	Maroon	No
45	1.2	Grey	No
48	1.1	Maroon	No
49	1.3	Maroon	No
39	1.4	Maroon	No
38	1.3	Maroon	Yes
55	1.4	Maroon	No
51	1.1	Maroon	No
43	1.2	Maroon	Yes
44	1.7	Maroon	No
45	1.6	Maroon	No
46	1.2	Maroon	Yes
48	1.3	Maroon	No

SPECIMENID	DELAYEDVILLOUSMATURITY
C13	absent
C14	absent
C21	absent
36C	absent
37C	absent
39C	absent
42C	absent
46C	N/A
52C	absent
61C	absent
62C	absent
75C	absent
77C	absent
83C	N/A
85C	absent
86C	absent
92C	absent
96C	absent

ACCELERATEDVILLOUSMATURITY	DISTALVILLOUSHYPOPLASIA
absent	absent
absent	absent
absent	N/A
absent	absent
absent	present
absent	absent
absent	present
absent	absent
N/A	present
N/A	present
absent	absent
absent	absent
absent	Present
absent	absent
absent	Present
absent	absent
absent	absent
absent	Present

VILLOUSEDEMA	IFPRESENTPERCENTAGEAFFECTED	VILLOUSNECROSIS
N/A	N/A	N/A
N/A	N/A	N/A
N/A	N/A	N/A
N/A	N/A	N/A
N/A	N/A	N/A
absent	N/A	absent
absent	N/A	absent
absent	N/A	absent
N/A	N/A	N/A
absent	N/A	absent
absent	N/A	absent
absent	N/A	absent
absent	N/A	absent
absent	N/A	N/A
absent	N/A	absent
absent	N/A	absent
N/A	N/A	N/A
absent	N/A	absent

IFYESPERCENTAG	SYNCYTIALKNOTS	IFPRESENTPERCENTAEAFFEC
N/A	present	25%
N/A	Absent	N/A
N/A	present	50%
N/A	present	25%
N/A	Absent	N/A
N/A	Absent	N/A
N/A	present	75%
N/A	absent	N/A
N/A	present	20%
N/A	absent	N/A
N/A	present	25%
N/A	present	N/A
N/A	present	25%
N/A	absent	N/A
N/A	present	30%
N/A	absent	N/A
N/A	Absent	N/A
N/A	present	25%

DETERMINEWHETHERINCREASEDORDECREASEDFORGESTATIONAL
decreased
N/A
normal
decreased
N/A
absent
normal
N/A
decreased
N/A
reduced
N/A
decreased
N/A
decreased
N/A
N/A
decreased

THICKENINGOFVILLOUSBASEMENTME	IFPRESENTVILLIPERCENTAGE
N/A	N/A
N/A	N/A
N/A	N/A
N/A	N/A
N/A	N/A
absent	N/A
absent	N/A
absent	N/A
N/A	N/A
absent	N/A
absent	N/A
absent	N/A
absent	N/A
absent	N/A
absent	N/A
absent	N/A
absent	N/A
absent	N/A

VILLOUSSTROMALFIBROSIS	IFPRESENTVILLIPERCENT/	VILLITIS	IFPRESENTPERCE
N/A	N/A	absent	N/A
N/A	N/A	absent	N/A
N/A	N/A	absent	N/A
N/A	N/A	present	N/A
N/A	N/A	absent	N/A
absent	N/A	N/A	N/A
absent	N/A	absent	N/A
absent	N/A	absent	N/A
N/A	N/A	absent	N/A
absent	N/A	N/A	N/A
absent	N/A	absent	N/A
absent	N/A	absent	N/A
absent	N/A	absent	N/A
absent	N/A	absent	N/A
N/A	N/A	N/A	N/A
absent	N/A	absent	N/A
N/A	N/A	absent	N/A
absent	N/A	present	N/A

INTERVILLOSITIS	IFPRESENTPROPORTIONOFINTI	FIBRINDEPOSITION
present	N/A	present
present	N/A	absent
absent	N/A	present
absent	N/A	absent
absent	N/A	absent
N/A	N/A	absent
absent	N/A	present
absent	N/A	present
absent	N/A	absent
absent	N/A	absent
absent	N/A	present
absent	N/A	present
N/A	N/A	absent
absent	N/A	absent
absent	N/A	absent
present	N/A	absent
absent	N/A	absent
N/A	N/A	absent

IFPRESENTPATTERNANDPROPORTIC	FETALINFLAMMATORYRESPONSE
N/A	present
N/A	present
N/A	absent
N/A	present
N/A	absent
N/A	N/A
N/A	absent
N/A	absent
N/A	absent
N/A	absent
N/A	absent
N/A	absent
N/A	N/A
N/A	present
N/A	N/A
N/A	present
N/A	present
N/A	present

MASSIVEHISTIOLYTICINTERVILLOUS	VILLOUSVASCULARITY
N/A	reduced
N/A	N/A
N/A	N/A
N/A	N/A
N/A	N/A
N/A	N/A
absent	N/A
absent	N/A
N/A	N/A
absent	N/A
N/A	increased
N/A	increased
N/A	N/A
absent	N/A
absent	N/A
N/A	increased
absent	N/A
absent	N/A

BASALVESSELWALLABNORMALITY	IFPRESENTWHICHONE
N/A	N/A
N/A	N/A
N/A	N/A
N/A	N/A
N/A	N/A
absent	N/A
absent	N/A
N/A	N/A
N/A	N/A
N/A	N/A
absent	N/A
absent	N/A
absent	N/A
absent	N/A
absent	N/A
N/A	N/A
absent	N/A
absent	N/A

Cord Abnormality 1	Cord Abnormality 2
Absent	N/A
N/A	N/A
Absent	N/A
Absent	N/A
Absent	N/A
Absent	N/A
N/A	N/A
N/A	N/A
N/A	N/A
N/A	N/A
Absent	N/A
Absent	N/A
N/A	N/A
Absent	N/A
N/A	N/A
Absent	N/A
N/A	N/A
Absent	N/A

FETALTHROMBOTICVASCULOPATHY	Membrane
Absent	N/A
Absent	N/A
present	N/A
N/A	extraplacental membrane with mild diffuse inflammato
N/A	extraplacental membrane within normal limits
N/A	N/A
N/A	membrane not captured
N/A	explacental membrane within normal limits
N/A	extraplacental membrane within normal limits
N/A	extraplacental membranes represened not adequate
present	extraplacental membranes within normal limit
present	N/A
N/A	extraplacental membrane within normal limits
Absent	N/A
Absent	extraplacental membrane not represented
Absent	extraplacental membrane has mild chronic inflammato
N/A	placental membrane with diffuse chronic inflammatory
Absent	extraplacental membrane exhibit dense chronic inflam

OTHERSIGNIFICANTFINDINGS

malaria pigment and hemosiderin noted,extraplacental membrane has diffuse mixed inflammatory infiltration of ma

extraplacental membrane exhibit diffuse acute infammatory infiltration,umblical cord not represented,diffuse intervi

extraplacental membrane not represented,partially presented umblical cord within normal limits, placental disk with

3 vessel cord with difusse mixed inflammatory infiltrate of polymorphonuclear cells,plasma cells and macrophages.

no whole umlical cord represented but within normal limits,terminal villi paucity and increased intervillus spaces. no

placental disk and extraplacental membrane within normal limits. cord not well represented

umblical cord not represented. placental disc bears reduced distal/terminal villi with abundant perivillous hermorha

3 vessel cord noted within normal limit. placental disk with perivillus fibrin deposition, foci of dystrophic calcification.

placental disk has villus paucity

placental disk has villus atrophy and paucity. areas of diffuse dystrophic calcification noted. no umbilical cord repre

3 vessel cord noted with thrombosis of all vessels.placental disk show occlusive thrombi in large stem vessels. term

extraplacental membrane within normal limits,3 vessel umbilical cord within normal limit.placental disk with some st

no umblical cord represented. placental disk has tiny elongated villi with increased intervillus spaces. no inflammat

extraplacental membrane and umblical cord have extensive chronic inflammatory infiltrate of lympho-plasmocyte a

umblical cord not represented. placental disk has increased villus paucity with increased intervillus spaces. villi are

the umbilical cord has mild lymphoplasmocyte infiltration.placental disk has chronic lympho plasmocyte intervillositis

umbical cord not represented.placental disk too tiny for diagnostic purpose.

no umbilical cord.tiny fragment of placental disk tissue show inflammatory cells within the villi majorly lymhocytes. p

Diagnosis 1
intervillositis, malaria pigment/hemosiderin, chorioamniositis
chorioamnionitis, intervillositis consistent with placental malaria
fetal thrombotic vasculopathy
chorioamnionitis, villitis of unknown etiology
distal villus hypoplasia
N/A
distal villus hypoplasia
perivillus fibrin consistent with maternal flow interaction
distal villus hypoplasia
distal villus hypoplasia and calcification
fetal thrombotic vasculopathy
fetal thrombotic vasculopathy
distal villuos hypoplasia
severe chorioanmionitis; severe funisitis
distal villus hypoplasia
choriamnionitis; funisitis
chorioanmionitis
Distal villous hypoplasia; chorioanminitis

Diagnosis 2
N/A
N/A
massive perivillous fibrin
funisitis, chorangiosis
arterial sclerosis
N/A
pigmentation of RBC's, infarction
N/A
N/A
N/A
villus choriongiosis
fibrin deposition. note;correlate with gestational age for the increased ynctitial knots
chorangiosis
placental malaria
N/A
intervillositis with malaria pigment
decidualitis
villitis[inflamation origin]

SPECIMEN ID	MEAN CROSS SECTIONAI	MEAN PERIMETER (UM)
C13	1438	148.89
C14	1064.81	123.08
C21	1516.13	143.59
C36	793.4	109.82
C37	1818.76	181.89
C39	893.28	115.14
C42	761.26	109.72
C46	1695.22	161.81
C52	1783.94	176.42
C61	813.68	115.41
C62	1185.08	138.97
C75	1124.54	126.74
C77	1734.47	176.36
C83	1559.24	153.45
C85	1573.49	160.06
C86	1618.31	158.92
C92	1242.78	142.16
C96	1159.37	138.08

MEAN DIAMETER (UM)	MEAN VOLUME DENSITY (PERCENTAGE)
48.77	67.27
41.04	80
47.65	64.24
39.84	77.57
63.92	21.21
40.75	62.42
39.56	44.84
53.97	67.87
62.13	48.48
42.69	67.27
47.02	71.51
44.73	73.93
65.32	18.18
55.06	73.93
56.18	26.66
53.15	74.54
50.12	60.6
45.97	47.27
